# Translation and psychometric testing of the Persian Version of nurses’ ethical decision-making in End-of-Life Care Scale

**DOI:** 10.1186/s12912-024-01981-2

**Published:** 2024-05-08

**Authors:** Erfan Pourshahri, Fateme Mohammadi, Habib Shareinia, Fozieh Abadi, Mostafa Bijani

**Affiliations:** 1 Student Research Committee, School of Nursing, Fasa University of Medical Sciences, Fasa, Iran; 2 Chronic Diseases (Home Care) Research Center and Autism Spectrum Disorders Research Center, Department of Nursing, Hamadan University of Medical Sciences, Hamadan, Iran; 3 Department of Medical-Surgical Nursing, School of Nursing, Gonabad University of Medical Sciences, Gonabad, Iran; 4 Department of Medical Surgical Nursing, School of Nursing, Fasa University of Medical Sciences, Fasa, Iran

**Keywords:** Ethical decision-making, Nurses, Psychometric testing, Palliative care

## Abstract

**Background:**

Ethical decision-making in end-of-life care is one of the most challenging aspects of healthcare: providing ethical care to the society is one of the most important responsibilities of healthcare professionals. In order to assess nurses’ ethical decision-making in end-of-life care, researchers need a specialized and comprehensive instrument which is sufficiently valid and reliable. The present study was conducted to translate and test the psychometric properties of the Persian version of Nurses’ Ethical Decision-Making in End-of-Life Care Scale (NEDM-EOLCS).

**Methods:**

This is a cross-sectional, multi-centric study with a methodological design The participants were selected via convenience sampling from five hospitals located in Iran. In total, 1320 nurses (660 for exploratory factor analysis and 660 for confirmatory factor analysis) participated in the study. The original NEDM-EOLCS was translated into Persian and subsequently the psychometric properties of the scale were assessed according to COSMIN criteria.

**Results:**

Exploratory factor analysis (EFA) showed the factor loading of the 55 items to be between 0.62 and 0.88, all of which were significant. Also, exploratory factor analysis showed that 3 factors (perceived professional accountability, moral reasoning/moral agency and moral practice) explained 74.51% of the variance. Confirmatory factor analysis (CFA) results verified the good fit of the data (a chi-square of 21.74, df = 7, *P* = 0.001) RMSEA = 0.01, CFI = 0.96, NFI = 0.95, and TLI = 0.97). The reliability of the scale was measured in terms of its internal consistency and the Cronbach’s alpha of the whole instrument was found to be 0.98.

**Conclusion:**

The Persian version of NEDM-EOLCS for nurses is sufficiently valid and reliable. Thus, this instrument can be used to measure nurses’ ethical decision-making in end-of-life care and identify the most effective strategies, e.g. educational interventions, to improve ethical decision-making skills in end-of-life care in these healthcare professionals as necessary.

## Introduction

Changes in the burden of diseases in the past century have led to the emergence of new, hard-to-treat, and chronic diseases in many societies today. Many individuals require advanced medical care at different times of their illnesses or lives [1]. Despite various physical and psychological complications caused by their illness, many patients continue to live, but they are deprived of a normal, active life and their existence depends on certain medical devices and special cardiopulmonary care. These patients are known as end-of-life cases [2]. Every year, approximately 56 million deaths occur worldwide, 85% of which happen in developing countries [3]. According to World Health Organization (WHO), approximately 56.8 million of the world’s population are in need of palliative care, 25.7 million of whom are in the last few years of their lives [4]. Many professional care providers and family caregivers are involved in caring for terminally ill patients, underscoring the significance of end-of-life care in healthcare systems [2]. Studies show that most patients are not willing to make decisions about their care in their end-of-life stage and most of the decisions related to Do-Not-Resuscitate (DNR) are made by their care providers or family members [5, 6]. Many other patients spend most of their time following various treatments to live longer, even if their chances of recovery are very little [7]. Yet, the number of patients who want to die in peace is noteworthy [7]. Since nurses are in closest proximity to patients and their families, they can explain the objectives of treatments and the status quo to the patients and their families and help them express their preferences [8]. Thus, they play a crucial part in improving the perspective of patients in the end-of-life stage [6].

Research shows that work overload, patients’ families’ unrealistic expectations, and insufficient training in end-of-life care impede nurses from understanding the needs of their patients and establishing an effective relationship with them [9]. The quality of end-of-life care depends on how nurses perceive their role as a care provider in this situation and how likely they are to take the right measures from their patients’ perspective [8]. Making the right decision in end-of-life care is usually very challenging [10], as it directly impacts the life and death of the patient [11]. Such ethical challenges as the uselessness of treatment, lack of doctors, encounters with dying patients, and shortage of workforce can influence decision-making for patients in end-of-life stage [10]. Failure to manage these ethical conflicts can expose nurses to great stress [12] and reduce the quality of care provided by them [13]; therefore, the process of ethical decision-making by nurses should be carefully examined. Ethical decision-making by nurses is a sequential process which includes professional responsibility and such ethical elements as ethical sensitivity, judgment, motivation and behavior, which, in turn, follow ethical reasoning [8]. A study by Goethals shows that this decision-making process cannot be regarded as an exclusively cognitive process as it is affected by personal factors, including personal values, experience, knowledge, and skill, and underlying values, including the opinions and expectations of one’s peers and family and the doctors [14]. According to, Botes when they are making ethical decisions, nurses do not exercise critical thinking, indicating that nurses are not sufficiently prepared to cope with ethical issues [15].

Studies report that nurses’ awareness of their ethical responsibilities in nursing care is increasing; however, they have difficulty in identifying ethical issues and determining a proper way to deal with them [14, 16]. Evaluation of ethical decision-making and its subscales depends on reliable and valid instruments designed in the context of different cultures [17, 18].

Crisham (1981), developed the nursing dilemma test (NDT): The nurses’ moral reasoning, decision-making, practical considerations, and familiarity with moral dilemmas were measured by this questionnaire. However, this tool covers only some ethical dilemmas in end-of-life care and may not address other aspects of ethical care and decision-making in patients at the end of life that require further investigation. Therefore, the need for an accurate, comprehensive, and dedicated tool to assess nurses’ level of ethical decision-making in end-of-life care is becoming increasingly apparent [19]. Huang (2020), developed and Psychometric properties of the Chinese of the End-of-Life Decision-Making and staff Stress Questionnaire. Although the instrument used in this study assesses aspects of ethical care and decision-making, including ethical sensitivity, it cannot comprehensively and specifically assess nurses’ ethical decision-making in end-of-life care. Therefore, there is a need for an accurate and comprehensive tool in this area [20].

A literature review reveals limited research on nurses’ ethical decision-making in end-of-life care in Iran over the past decade (2013–2023). While some studies have explored ethical decision-making, such as, Khaghanizadeh’s, et al. (2023) investigation of the effect and comparison of training in ethical decision-making through lectures and group discussions on moral reasoning, moral distress and moral sensitivity in nurses [21] and, Jamshidi’s, et al., study of the effects of an ethical empowerment program on critical care nurses’ ethical decision-making [22], However, the studies did not employ a comprehensive tool to measure nurses’ ethical decision-making in end-of-life care, which highlights the need for a valid and specific tool to assess the level of nurses’ ethical decision-making in this area.

In 2010, Kim et al. developed and tested the Nurses’ Ethical Decision Making around End of Life Care Scale (NEDM-EOLCS) to assess nurses’ ethical decision-making skills in caring for dying patients. The scale consists of 55 items which are scored on a 6-point Likert scale and addresses perceived professional accountability (28 items), moral reasoning/moral agency (13 items), and moral practice (14 items), which makes it superior to a one-dimensional instrument. Higher scores indicate better ethical decision-making skills [23].

Valid instruments developed based on ethical perspectives, cultural variations, and distinct domains and variables are essential to enhancing knowledge about ethical decision-making in end-of-life care. Such instruments can contribute to an understanding of what is needed to improve nurses’ competence in caring for patients who require end-of-life care. So far, there has not been a specialized and comprehensive tool suitable for use in the cultural context of Iran for evaluating nurses’ ethical decision-making in end-of-life care. Given the paramount importance of assessing and evaluating the ethical decision-making capabilities of healthcare professionals entrusted with the care of end-of-life adolescent patients, the absence of a valid and reliable tool in the Iranian context presents a significant challenge. The design and development of tools, particularly psycho-cognitive scales, are demonstrably influenced by a complex interplay of factors, including beliefs, values, cultural norms, and religious frameworks. Consequently, the application of such instruments to a population or society with a distinct cultural identity necessitates a rigorous process of psychometric adaptation tailored to the specific sociocultural context. In addition, cultural differences between different societies necessitate the translation and psychometric evaluation of NEDM-EOLCS in different cultures. Accordingly, the present study was conducted to translate and measure the psychometric properties of the Persian version of NEDM-EOLCS.

## Methods

### Research design

Conducted from February 2022 to August 2022, the present study used a cross-sectional, multi-centric study with a methodological design to evaluate nurses who were in practice in hospitals located in Fars Province, south of Iran. After obtaining the necessary ethical code and permits, and coordinating with the hospital president and manager, the researcher entered the hospital wards during various work shifts. The researcher then provided the nurses of each ward with clear explanations about the purpose and methodology of the research. If the nurses agreed to participate in the study, they were given the questionnaires. The researcher provided necessary explanations to the nurses if they had difficulty understanding the questionnaire. Finally, the researcher collected the completed questionnaires from the nurses.

The researchers used COSMIN (Consensus-based Standards for the selection of health Measurement Instruments) criteria to measure the psychometric properties of NEDM-EOLCS: content validity, reliability (internal consistency and stability) and construct validity (exploratory factor analysis and confirmatory factor analysis) [24].

### Sample size, inclusion and exclusion criteria

For evaluation of the psychometric properties of the scale, the researchers used the number of inventory sections to determine sample size, which was set at 10 subjects per item [25]. However, in the present study, about 12 respondents per item were recruited to enhance the accuracy of exploratory and confirmatory factor analyses. The participants were selected via convenience sampling from five hospitals located in Iran. In total, 1320 nurses (660 for exploratory factor analysis and 660 for confirmatory factor analysis) participated in the study. The nurses recruited for exploratory factor analysis were different from the nurses recruited for confirmatory factor analysis. The inclusion criteria were: being willing to participate in the study and having at least one year’s experience of professional practice. All the participants completed the informed consent form of the study. The participants who were not willing to continue participating for any reason or did not complete the questionnaires were excluded.

### Nurses’ ethical decision-making in end-of-Life Care Scale (NEDM-EOLCS)

Developed and tested by Kim et al. [23], the NEDM-EOLCS measures nurses’ ethical decision-making skills in caring for dying patients. The scale consists of 55 items and three subscales: perceived professional accountability (28 items), moral reasoning and moral agency (13 items), and moral practice (14 items). The study by Kim et al. showed that the Korean version of NEDM-EOLCS was adequately reliable: they reported that the Cronbach’s alpha coefficients of the 3 subscales of the scale were between 0.84 and 0.94 and the intra-class correlation coefficient (ICC) of the whole scale was 0.90.

### Translation and cultural adaptation

The questionnaire was translated after the corresponding author (MB), obtained permission from the developers of the scale. Translation was carried out based on the translation and cross-cultural adaptation guidelines by Beaton et al. [26]. The English version of NEDM-EOLCS was translated into Persian using the forward-backward approach in six stages: (1) in the forward translation stage, two bilingual translators, native speakers of English and Persian and also familiar with the Iranian culture, translated the English script into Persian; (2) then the two translations were examined by two other translators and the research team and synthesized into a single script; (3) in the backward stage, a bilingual translator translated the Persian version of the questionnaire, from the previous stage, from Persian into English; (4) in the expert committee stage, a group consisting of instrument development experts, nurses, doctors and translators reviewed the translated versions of the questionnaire from the previous stages and unanimously agreed on a final version; (5) next, the final version was evaluated by 50 nurses who were randomly selected and asked to assess the Persian scale (their feedback was used to revise and improve the scale); (6) finally, the psychometric properties of NEDM-EOLCS were measured using COSMIN criteria, including face validity, content validity, reliability (internal consistency and stability) and construct validity.

### Face validity

#### Qualitative face validity

In 15 face-to-face interviews with nurses and nursing and instrument development experts, the difficulty level, suitability, and relevance of the items on NEDM-EOLCS were evaluated.

### Quantitative face validity

For quantitative face validity, 15 nurses were asked to rate the significance of each item on the scale on a 5-point Likert scale (1 = Not important at all to 5 = Very important). Subsequently, the impact score of each item was calculated and the items whose impact score was greater than 1.5 were retained [27].

### Content validity

#### Qualitative content validity

In this stage, 15 nursing and instrument development experts (ten of the experts had a PhD in nursing and five had a master’s degree) and 15 nurses who were practicing in hospitals were provided with the translated version of NEDM-EOLCS. They were asked to assess the scale items in terms of syntax, phrasing, clarity, and compatibility with the Iranian culture.

### Quantitative content validity

Content validity ratio (CVR) was evaluated by a team of experts who were asked to rate the relevance and necessity of the items on a 3-point Likert scale (1 = Unnecessary to 3 = Necessary). The content validity of each item was calculated in this manner. As for content validity index (CVI), 30 subjects were provided with the revised version of NEDM-EOLCS and asked to score each item in terms of simplicity, clarity, and relevance on a 4-point Likert scale (1 = Irrelevant to 4 = Relevant) [28]. Next, the CVI of each item and the total CVI of the scale were calculated. In the present study, CVRs of greater than 0.33 and CVIs of greater than 0.8 were considered appropriate.

### Construct validity (exploratory factor analysis and confirmatory factor analysis)

The purpose of exploratory factor analysis (EFA) is to assess construct validity to ensure that an instrument actually measures what it has been designed to measure. In the present study, EFA was executed using Varimax rotation. To achieve optimum construct, the researchers set eigenvalues at greater than 1 and factor loading at greater than 0.4. Sampling adequacy was tested using Kaiser–Meyer–Olkin (KMO) and Bartlett’s tests. KMO values should be above 0.7 and the Bartlett’s test value should be less than 0.05 (*p* < 0.05) [29]. If the factor loading of an item was less than 0.4, that item was eliminated. In this study, 12 nurses were selected per item; therefore, 660 nurses participated in the evaluation of the exploratory factor analysis of the scale. The results showed that the factor loading of each item was greater than 0.4; thus, none of the items was eliminated.

Confirmatory factor analysis was implemented using 660 practicing nurses other than the ones who were involved in the exploratory factor analysis stage. The researchers used AMOS (v. 21.0) for confirmatory factor analysis. Several indexes were used to measure the usefulness of the model: a Goodness of Fit (GFI) of greater than 0.90, a root mean square error of approximation (RMSEA) of less than 0.08, a Tucker Lewis Index (TLI) acceptable at greater than 0.90, and a comparative fit index (CFI) acceptable at greater than 0.9026 [30].

### Reliability

The reliability of NEDM-EOLCS was determined using the methods for measuring internal consistency (Cronbach’s alpha) and stability (test-retest). Cronbach’s alpha was calculated for 660 subjects, and values of above 0.7 were regarded as acceptable [31]. As for measuring internal consistency via the test-retest method, the intraclass correlation (ICC) between the data collected from 200 practicing nurses with a two-week interval was calculated. If the ICC of an instrument is greater than 0.80, it indicates that the consistency of the instrument is satisfactory [32].

### Ethical considerations

The principles of the revised Declaration of Helsinki, which is a statement of ethical principles that direct physicians and other participants in medical research involving human subjects, were considered in all parts of the present study. All participants signed the informed consent to participate in the study. The participants were assured that all their personal information would remain confidential, and that they were free to withdraw at any stage of the study. We provided them with sufficient information as to the anonymity and confidentiality of their information. Moreover, the Research Ethics Committees of Fasa University of Medical Sciences, Fars, Iran approved the study with the code of IR.FUMS.REC.1401.216.

## Results

The participants of the present study consisted of 1320 nurses who were practicing in hospitals located in Fars province, south of Iran. The majority of the nurses were female (65%), the nurses’ ages ranged from 21 to 52 years, with a mean of 39.05 ± 6.89 years, and their average work experience was 10.36 ± 5.44 years. The other demographic characteristics of the participants are shown in Table 1.

**Table 1:** Frequency distribution of demographic characteristics (*n* = 1320)

Variable		*N*	%
Gender	Male	462	35
	Female	858	65
Marital status	Unmarried	396	30
	Married	924	70
Education level	Bachelor’s degree in nursing	850	64.40
	Master degree in nursing	450	34.1
	PhD degree in Nursing	20	1.5
Ward	Surgical	100	7.57
	Internal	148	11.21
	I.C.U	140	10.60
	C.C.U	130	9.84
	Post.C.C.U	80	6.06
	Emergency	300	22.72
	N.I.C.U	110	8.33
	Pediatric	80	6.06
	Dialysis	60	4.54
	Oncology	84	6.36
	Nurology	88	6.66

### Qualitative content validity

#### Face validity

At this stage of the study, the nurses and nursing and instrument development experts who participated in the study stated that all the items on the scale were simple, clear, and relevant to the subject of the study. Moreover, the impact factor of all the items was above 1.5.

### Quantitative content validity

In qualitative evaluation of content validity, 30 of the nurses suggested that six items (items 5, 7, 21, 23, 27, and 48) should be revised to become more clear and comprehensible. After being revised, the items in question were reexamined and verified by a panel of experts. CVR was calculated based on the experts’ rating of the necessity of the items. According to Lawshe table, the acceptable value of CVR is 0.33 and above. The CVR of all the items on NEDM-EOLCS ranged from 0.79 to 1; thus, none of the items was eliminated. The CVI of each item was also calculated and was found to range from 0.89 to 1. The SCVI/Average of the scale equaled 0.97. Finally, the Modified Kappa Scale Content Validity Index/Average was determined to be 0.89, respectively.

### Construct validity

**Fig. 1:**
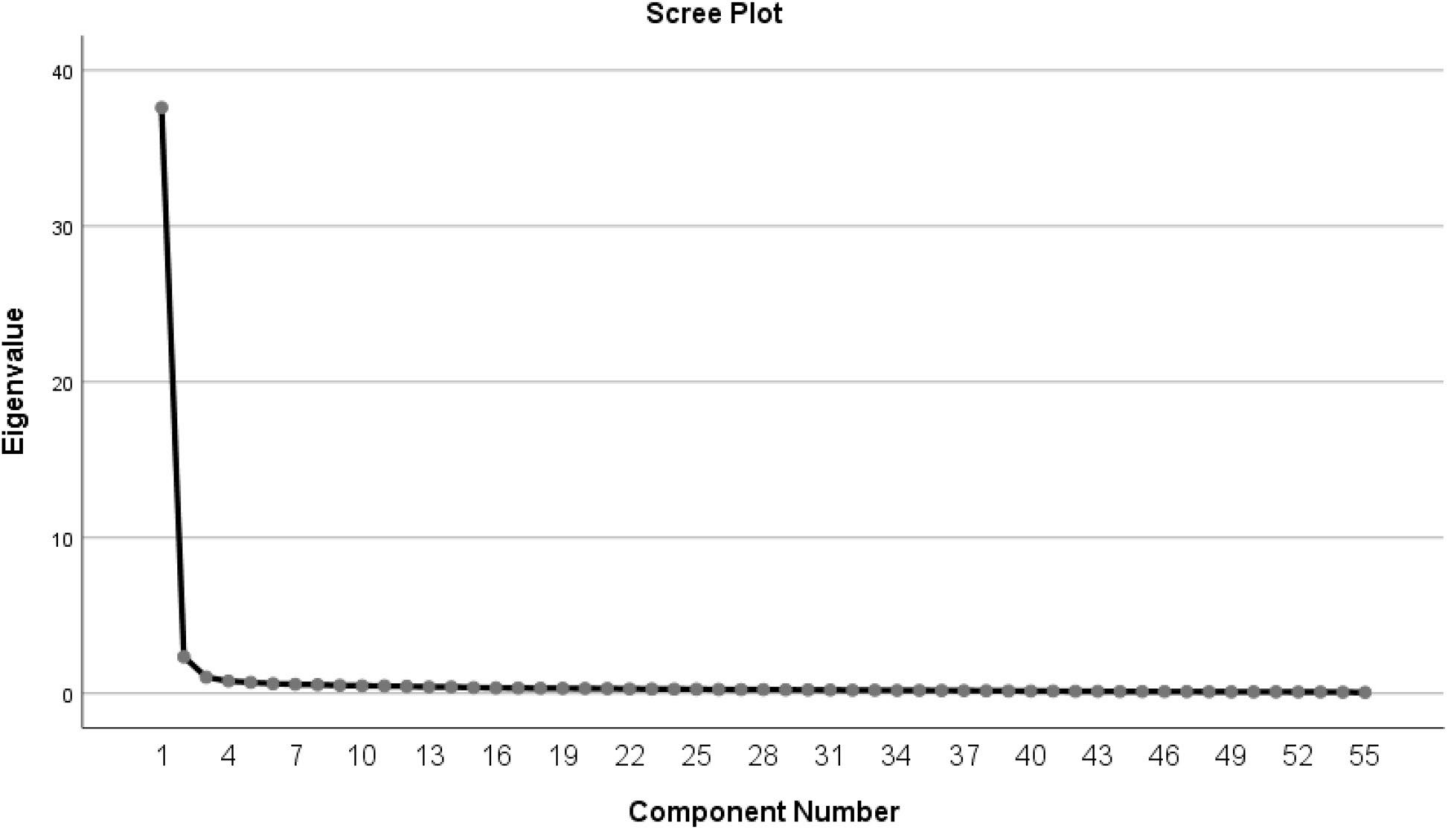
Scree plot of exploratory factor analysis for Persian Version of Nurses’ Ethical Decision-Making in End-of-Life Care Scale (NEDM-EOLCS)

 The first step in exploratory factor analysis is determining the value of KMO. The KMO of the present scale was found to be 0.985, which confirmed sampling adequacy for analysis. The factor loading of all the items was above 0.50; thus, none of the items was eliminated. The factor analysis results showed that three factors explained 74.51% of the total variance of the scale (χ2 = 32763.483; *P* < 0.001). The scree plot confirmed three factors for the scale (Fig. 1). Also, the results showed that the factor loading of the items ranged from 0.55 to 0.84 (the factor loadings are shown in Table 2).

**Table 2:** Varimax factor loadings of the items of the instrument

Factors’ names	Item	Factor loading
**Component 1: Perceived professional accountability at the end of life**	I must ensure that patients who have a do-not-resuscitate order still receive basic nursing care.	0.62
	Nurses are responsible for their own practice actions.	0.89
	Routine nursing and medical procedures have ethical implications for individual patients.	0.78
	Nurses are responsible for providing adequate information about the patient’s care.	0.73
	It is important that I remain focused on the responsibility I have toward my patient.	0.82
	Nurses are responsible for providing the best care for patients at the end of life.	0.76
	When I feel a connection with the patient, I am more likely to act to meet their needs.	0.75
	Nurses are responsible for ensuring that a patient’s suffering is relieved at the end of life.	0.75
	Nurses are responsible for advocating that a patient’s individual needs are met.	0.78
	Nurses are responsible for assisting patients to make the best healthcare decision.	0.74
	It is my professional responsibility to get my patients needs met even when this is difficult.	0.77
	When patients and/or their family are thankful for my actions, it encourages me to persist in getting them what they need.	0.69
	The nurse should put the patient’s safety as the first priority when he/she experiences a conflict with others over the patient’s care.	0.75
	All nursing action for a patient should be informed by knowledge, skill, experience, and an understanding of that patient’s individual need.	0.75
	Nurses are responsible for encouraging the patient to be involved in the process of his/her care if the patient is capable.	0.76
	My actions make a difference to the patient who is facing the end of life.	0.75
	Nurses are responsible for assisting patients to receive hospice or palliative care when invasive interventions are no longer desired or effective.	0.75
	A nurse should refuse to participate in activities that are harmful to the patient.	0.76
	It is important that I am sensitive to the individual needs of patients and their family.	0.65
	The support of my colleagues helps to keep me focused on getting my patient’s needs met.	0.62
	Nurses should ensure patients receive good care even if the patient is difficult or undesirable.	0.71
	Nurses are responsible for recognizing the unethical practice of others and doing something about it.	0.69
	Nurses are responsible for advocating that the patient gets what he/she needs even when another nurse, doctor, or family member disagrees with the patients’ considered wishes or desire.	0.79
	I recognize what the other health professionals’ roles and their responsibilities are.	0.66
	Nurses should use their clinical judgment in deciding whether a treatment or intervention is appropriate for a patient.	0.66
	The nurse should support the patient’s reasoned decision to accept or refuse treatment.	0.74
	My personal beliefs and values can make me biased toward a particular course of action so I try to understand what these are before acting.	0.62
	It is meaningful for me to ensure that I care for a patient who is facing the end of life.	0.69
Component 2: Moral reasoning and moral agency	I actively engage in ethical conflict during the end of life care and persist until the patient gets what he/she needs.	0.68
	I feel strongly that I must try to resolve an ethical problem even if this is risky for me.	0.73
	I can separate out the barriers to good care in an ethical conflict.	0.71
	I can identify when an EOL decision is being made that is not in the interests of the patient.	0.67
	When institutional policies related to end of life practices are inappropriate, I use current evidence to try to change them.	0.64
	I step back from ethical conflicts and try to think through the issues to find a solution.	0.72
	I am able to describe the ethical aspects of a difficult patient situation.	0.69
	I confirm the patients’ wishes or preferences regarding do-not-resuscitate decisions made by family members.	0.73
	I know who to go to get help in thinking through a difficult situation.	0.68
	I try to ensure that the patient and his/her family are satisfied with their decisions making.	0.70
	I feel compelled to act on behalf of my patients when I see they are not getting their needs or wishes met.	0.62
	I confront other healthcare providers when their actions are unethical and might cause harm.	0.65
	When I am tired or upset, I am still able to focus on meeting my patient’s needs in a problematic situation.	0.69
Component 3: Moral practice at the end of life	I try to help patients find meaning in their condition when they are facing the end of their lives.	0.64
	I seek out available and current empirical evidence to provide appropriate end of life care to patients.	0.71
	I try to be a comforting presence for the patient who is at the end-of-life even when he/she does not need hands-on care.	0.73
	I try to tailor care to a patient’s individual need.	0.68
	I try to persuade other healthcare professionals and the patients’ family to honor the patient’s wishes when they are acting contrary to what the patient wants.	0.73
	I try to help patients at the end-of-life repair problem relationships they have with important family members or friends.	076
	I use knowledge of what actions I would want for my family members to help provide care for the patients.	0.74
	I ask the patient what he/she needs related to the dying process. I provide appropriate information about the purposes and goals of withdrawing or withholding treatment.	0.70
	I provide appropriate information about the purposes and goals of withdrawing or withholding treatment.	0.73
	I try to understand what the patient’s preference regarding end of life care is and to advocate for this to be heard by those making the decisions.	0.69
	I try to mediate between the patient’s family and other healthcare providers when there is conflict about the goals of care.	0.69
	I try to provide education to the patient and family about the purpose of any technology or therapies being used.	0.70
	I encourage the patient’s family to be with the patient for the in all hours.	0.64
	I try to meet with the patient’s family regularly and answer their questions.	0.68

### Confirmatory factor analysis

**Fig. 2:**
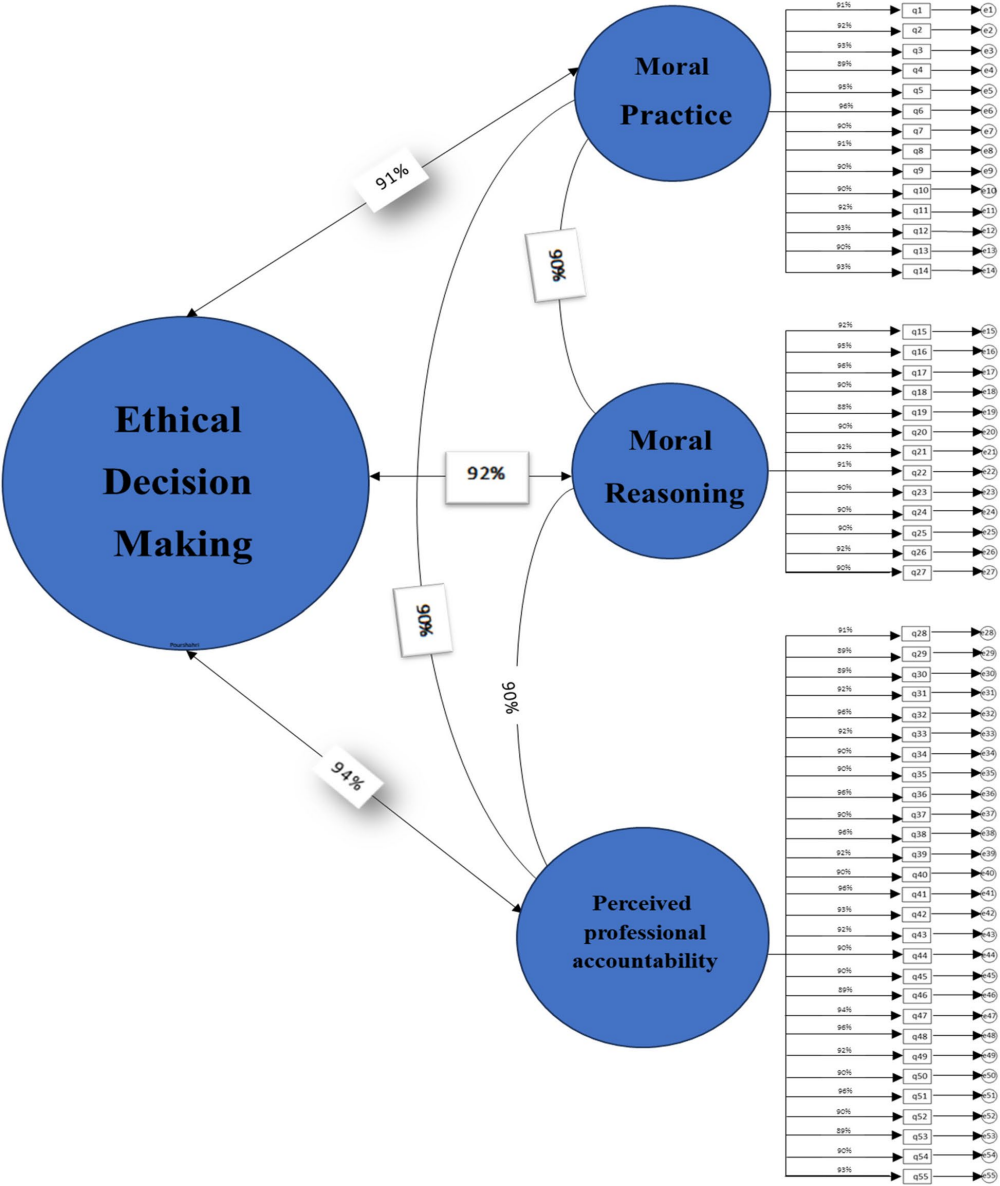
Confirmatory factor analysis for Persian Version of Nurses’ Ethical Decision-Making in End-of-Life Care Scale (NEDM-EOLCS)

The confirmatory factor analysis resulted in a model with three factors: professional accountability (28 items), moral reasoning (13 items), and moral practice (14 items). The correlation of factors 1, 2, and 3 with the entire scale was 0.94, 0.92, and 0.91, respectively. In addition, a chi-square of 21.74, df = 7, *P* = 0.001) indicated good fitness of the model. The Goodness of Fit Index (GFI) equaled 0.94, showing that the one-dimensional model of PTES constructs fitted well in the present study. The other indices measured in this model were the following: RMSEA = 0.01, CFI = 0.93, NFI = 0.95, and TLI = 0.94. All the tested indices confirmed that the extracted model fitted well (Fig. 2).

### Reliability (internal consistency and stability)

#### Internal consistency

The internal consistency of the scale equaled a Cronbach’s alpha of 0.98, indicating that the scale had satisfactory internal consistency (Table 3).

**Table 3:** Cronbach’s alpha of subscales and the entire Persian Version of Nurses’ Ethical Decision-Making in End-of-Life Care Scale (NEDM-EOLCS)

Factors	Subscale	Items	Cronbach’s alpha
1	Perceived professional accountability at the end of life	28	0.98
2	Moral reasoning and moral agency	13	0.96
3	Moral practice at the end of life	14	0.97
Entire Questionnaire		55	0.98

#### Stability

The intra-class correlation coefficient across the 55 item-instrument was 0.92, which indicates the appropriate internal consistency of this questionnaire (Table 4).

**Table 4:** Intraclass correlation coefficient (ICC) values for the domains of the Persian Version of Nurses’ Ethical Decision-Making in End-of-Life Care Scale (NEDM-EOLCS)

Factor	Dimensions	Mean ± SD	ICC	Confidence interval	*P* -value
1	Perceived professional accountability at the end of life	137.52 ± 23.79	0.94	0.89–0.96	*p* < 0.05
2	Moral reasoning and moral agency	50.96 ± 10.93	0.97	0.877–0.98	*p* < 0.05
3	Moral practice at the end of life	54.90. ±11.64	0.88	0.81–0.97	*p* < 0.05
Entire Questionnaire (Total)			0.92	0.83–0.93	*p* < 0.05

### Determination of the ease of use of the questionnaire

To determine the ease of use of the questionnaire, the researchers measured the average time required to complete the questionnaire, which was found to be nine minutes (the range was from 8 to 10 min). The rate of nonresponse was less than 5%.

## Discussion

The present study was conducted to translate and evaluate the psychometric properties of a Persian version of NEDM-EOLCS for clinical nurses in Iran. The findings of the study showed that, as with the original version of the scale, the Persian version of NEDM-EOLCS was sufficiently valid and reliable. Evaluation of the face validity of the scale found that all the 55 items had an impact score of greater than 1.5; thus, none of the items was eliminated. Evaluation of the content validity of the scale showed that the CVR of the items ranged from 0.76 to 1, which is considered a satisfactory value [27]. The I-CVI of the scale was found to be between 0.80 and 1, and S-CVI was a satisfactory 0.94 [28]. In their study, Kim et al. did not measure the face validity and content validity of the scale, which is one of the strengths of the present study.

In the present study, the results of exploratory factor analysis showed that 3 factors explained 74.51% of the variance and the factor loading of the items ranged from 0.62 to 0.89, which is a satisfactory level. Similarly, Kim et al. reported that the results of exploratory factor analysis showed that the 3 subscales of the Korean version of NEDM-EOLCS accounted for 44.50% of the variance and the factor loading of the items was between 0.57 and 0.88, which is considered satisfactory [23]. In confirmatory factor analysis, the average extracted variance values were 0.72 to 0.87, and the model fitting indexes were all in an acceptable range.

The results of the study showed that the Persian version of NEDM-EOLCS possesses a satisfactory degree of reliability: The Cronbach’s alpha of the 3 subscales of the scale ranged between 0.96 and 0.98, and the Cronbach’s alpha of the whole instrument was found to be 0.98. Moreover, the intra-class correlation coefficient (ICC) of the entire scale was a satisfactory 0.92 [31]. Similarly, the study by Kim et al. showed that the Korean version of NEDM-EOLCS was adequately reliable: they reported that the Cronbach’s alpha coefficients of the 3 subscales of the scale were between 0.84 and 0.94 and the intra-class correlation coefficient (ICC) of the whole scale was 0.90. The present study investigated the presence of ceiling and floor effects within the newly developed questionnaire administered to nurses. These effects, if unaddressed, can significantly compromise the reliability of a measurement tool. In essence, the absence of ceiling and floor effects prevents the accurate evaluation of individuals at the extreme ends of the scoring spectrum, consequently diminishing the overall reliability of the instrument. Notably, prior research has failed to report any data pertaining to the ceiling and floor effects associated with existing tools in this domain.

Mohammadi, et al. (2024) developed a novel instrument to assess the moral intelligence of healthcare professionals specifically within the high-pressure environment of the cardiac operating room in Iran. This scale, comprised of 30 items, operationalizes the construct of moral intelligence through three distinct dimensions: moral sensitivity, moral commitment, and moral courage. The instrument demonstrates evidence of both face and content validity, ensuring that the items accurately reflect the intended construct and are relevant to the target population. Additionally, exploratory validity analyses reveal item factor loadings ranging from 0.608 to 0.923, indicating a strong association between individual items and their underlying dimensions. Furthermore, confirmatory factor analysis provides support for the hypothesized structure of the scale, and reliability estimates for each of the three dimensions range from 0.93 to 0.95, exceeding established thresholds for acceptable internal consistency. Although this study addressed aspects of ethical care, the instrument designed in this study examines moral intelligence in operating room personnel and is therefore not suitable for Nurses’ Ethical Decision-Making in End-of-Life Care [33]. Asahara et al. (2013) developed a multidimensional scale to evaluate moral competence among nurses in the Japanese home care setting. The instrument consists of 45 items and measures moral competence through five dimensions: moral sensitivity, moral judgment, moral motivation, moral personality, and moral decision-making. The scale demonstrates evidence of face and content validity, ensuring that the items accurately reflect the intended construct and are relevant to the target population of home care nurses. Exploratory factor analysis further reveals moderate to strong associations between individual items and their underlying dimensions, with item factor loadings ranging from 0.41 to 0.93. Additionally, confirmatory factor analysis supports the hypothesized structure of the scale. Reliability estimates for each of the five dimensions range from 0.78 to 0.93, which exceeds the established thresholds for acceptable internal consistency. While this study addressed aspects of ethical decision-making, the instrument designed in this study examines the moral competence of nurses in home care and is therefore not specifically and comprehensively tailored to the assessment of Nurses’ Ethical Decision-Making in End-of-Life Care [34].

### Limitations

The target population of the present study was nurses, and nursing students were not included. It is suggested that future studies explore nursing students’ views as well. Also, in view of cultural differences between different countries, it is suggested that NEDM-EOLCS be translated and tested in other countries. Since the scale was originally developed in Korea and evaluated only in that country, in the present study, the researchers could compare their findings to Kim’s study only, which is one of the limitations of the study. In the present study, long questionnaires yield higher Cronbach’s alpha values due to the increased number of items. This is because having more items often leads to higher inter-item correlation, and consequently, greater internal consistency. However, it is possible that participants may have chosen similar options for various reasons, such as social desirability bias or acquiescence bias. This factor should be considered as a limitation, and its implications for the interpretation of the Cronbach’s alpha value should be acknowledged.

### Strengths

A wide range of nurses participated in the present study and the scale was evaluated comprehensively. However, in the Korean study by Kim et al., where the scale was first developed, content and face validity were not measured and confirmatory factor analysis was not executed.

## Conclusion

The Persian version of NEDM-EOLCS is sufficiently valid and reliable. Thus, nurse managers can use this scale to measure nurses’ ethical decision-making in end-of-life care and identify the most effective strategies to improve ethical decision-making skills in nurses.

## Data Availability

The datasets generated and/or analysed during the current study are not publicly available due to the necessity to ensure participant confidentiality policies and laws of the country but are available from the corresponding author on reasonable request.

## Abbreviations

NEDM-EOLCS: Nurses’ Ethical Decision-Making in End-of-Life Care Scale
COSMIN: Consensus-based Standards for the selection of health Measurement Instruments
